# Synthesis and Biological Activity of *N*-Sulfonyltripeptides with C-Terminal Arginine as Potential Serine Proteases Inhibitors

**DOI:** 10.1007/s10989-012-9338-4

**Published:** 2012-12-02

**Authors:** Agnieszka Markowska, Magdalena Bruzgo, Ewa Gorodkiewicz, Arkadiusz Surażyński

**Affiliations:** 1 Department of Organic Chemistry, Medical University of Bialystok, Kilinski 1 Str., 15-089 Białystok, Poland; 2 Department of Instrumental Analysis, Medical University of Bialystok, Białystok, Poland; 3 Department of Electrochemistry, Institute of Chemistry, University of Bialystok, Białystok, Poland; 4 Department of Medicinal Chemistry, Medical University of Bialystok, Białystok, Poland

**Keywords:** Urokinase inhibitor, Serine proteases inhibitor, Amidolytic activity, Cancer cells activity, Surface Plasmon Resonance Imaging

## Abstract

Tripeptides of the general X-SO_2_-d-Ser-AA-Arg-CO-Y formula, where X = *α*-tolyl, *p*-tolyl, 2,4,6-triisopropylphenyl; AA = alanine, glycine, norvaline and Y = OH, NH-(CH_2_)_5_NH_2_ were obtained and tested for their effect on the amidolytic activities of urokinase, thrombin, trypsin, plasmin, t-PA and kallikrein. The most active compound towards urokinase was PhCH_2_SO_2_-d-Ser-Gly-Arg-OH with K_i_ value 5.4 μM and the most active compound toward thrombin was PhCH_2_SO_2_-d-Ser-NVa-Arg-OH with K_i_ value 0.82 μM. The peptides were nontoxic against porcine erythrocytes in vitro. PhCH_2_SO_2_-d-Ser-Gly-Arg-OH showed cytotoxic effect against DLD cell lines with IC_50_ values of 5 μM. For the highly selective determination of the interaction of some of the synthesised acids of tripeptides with urokinase and plasmin the Surface Plasmon Resonance Imaging sensor has been applied. These compounds bind to urokinase and plasmin in 0.05 mM concentration.

## Introduction

The proteolytic degradation of the extracellular matrix is essential for the processes of tissue remodelling. These processes take place in a number of distinct physiological events in the healthy organism such as matrix degradation, cell motility, angiogenesis and wound healing, as well as in the critical mechanisms in tumor invasion and metastasis. The plasminogen activation system has an important role as a proteolytic cascade in these degradation reactions (Irigoyen et al. 1999). The enzymatic system consists of urokinase receptor (uPAR), urokinase plasminogen activator (uPA) and plasminogen activator inhibitors: type-1 (PAI-1) and type-2 (PAI-2). uPAR is a cell membrane-anchored binding protein. uPA accumulates plasminogen activation activity at cell surfaces (Ossowski and Aguirre-Ghiso 2000). uPA is the primary cellular activator which converts the pro-enzyme plasminogen into its active form plasmin (Irigoyen et al. 1999). Plasmin is a broad spectrum serine protease which dissolve fibrin blood clots, activates metalloproteinases, degrades basement membrane and many blood plasma proteins (Longstaff and Thelwell 2005).

Numerous studies have shown the relationship between the level of expression of the uPA enzyme system and the poor prognosis in brain, colon, stomach, ovary, breast and kidney cancers (Duffy 2004; Sidenius and Blasi 2003; Dass et al. 2008). Urokinase has become an attractive therapeutic target in a variety of tumor.

Urokinase inhibitors are divided into 3 classes: inhibitors of the uPA–uPAR interaction, inhibitors of the uPA–PAI-1 interaction and inhibitors of uPA proteolytic activity.

uPA–uPAR interaction cyclic or linear inhibitors are based on a specific amino acid sequence which comes from the growth factor domain (GFD) of urokinase. These peptides contains from 10 to 32 amino acids from this region of uPA (Ploug et al. 2001; Goodson et al. 1994).

Corvas has described several substituted phenylpropionic acid derivatives as effective PAI-1 antagonists (Tamura et al. 2000). High levels of PAI-1 have been suggested as one of the factors that contribute a poor prognosis for cancer patients (Binder and Mihaly 2008).

Many small molecules have been reported to inhibit the proteolytic activity of uPA. These peptide-based compounds include the central aromatic or heteroaromatic (thiophen, indole, pyridine) core (Klinghofer et al. 2001; Schweinitz et al. 2004; Rockway and Giranda 2003). The ring is substituted for a basic moiety, either an amidine or a guanidine, which is essential to form a salt bridge with Asp189 of uPA.

The number of reports concerning peptides as urokinase inhibitors is limited. *N*-*α*-*p*-toluenesulphonyl-l-arginine methyl ester, ε-aminocaproic acid and its derivatives or natural leupeptin and its derivatives are known as inhibitors of urokinase (Rockway et al. 2002; Aoyagi et al. 1969). Glu-Gly-Arg-CH_2_Cl is a synthetic inhibitor which inhibits urokinase with K_i_ value 5 μM and alkylates the active-site histidine of urokinase (Lijnen et al. 1987). But the most active peptide uPA inhibitor is phenethylsulfonyl-d-Ser-Ala-Arg-H with IC_50_ value of 3.1 nM (Tamura et al. 2000).

On the basis of structure–activity literature data, we have recently designed a series of peptide analogs as urokinase inhibitors. The first series was H(Ac)-d-Ser-Ala-(Gly)-Arg-OH(NH_2_) (Markowska et al. 2008) and the most active inhibitor of urokinase was H-d-Ser-Gly-Arg-OH with an IC_50_ value of 1 mM. The second series of peptide analogs of arginine we synthesized were the peptides of the general formula H-d-Ser-Ala-Arg-NH-X (Markowska et al. 2010), where X = (CH_2_)_n_-NH_2_, *n* = 2–9, (CH_2_)_m_–OH, *m* = 2–4. H-d-Ser-Ala-Arg-NH-(CH_2_)_5_-NH_2_ inhibited urokinase with a K_i_ value of 6.3 μM. The third series of compounds with D-Ser-Ala-Arg sequence were *N*-sulfonylamides peptides (Markowska et al. 2010). 2,4,6-triisopropylphenyl-SO_2_-d-Ser-Ala-Arg-OH was the most selective inhibitor of urokinase and *α*-tolyl-SO_2_-d-Ser-Ala-Arg-OH was the most active inhibitor of uPA with a K_i_ value of 24 μM.

We inserted aliphatic amino acid in the other group of synthesised peptides in P2, and the most active compound towards urokinase was H-d-Ser-NVa-Arg-OH with a K_i_ value 0.85 μM (Markowska et al. 2011). Afterwards, we synthesised peptides with non-code aromatic and cyclic amino acid in P2 (Markowska et al. 2012) but this class of peptides was not the inhibitors of urokinase.

The purpose of this paper was the synthesis and examination of the amidolytic activity of 17 novel peptides against urokinase, thrombin, trypsin, plasmin, t-PA and kallikrein. The peptides had the general X-SO_2_-NH-d-Ser-AA-Arg-OH(NH-(CH_2_)_5_-NH_2_) formula, where X = *α*-tolyl, *p*-tolyl, 2,4,6-triisopropylphenyl and AA = Ala, Gly, NVa (Table 1.). We tested the hemolytic activity of the peptides against porcine erythrocytes and the antitumor activity against the following human cancer cells: colorectal adenocarcinoma tumor DLD, standard MCF-7 and estrogen-independent MDA-MB-231. We also examined the interaction of some of the synthesised acids of tripeptides with urokinase and plasmin through a Surface Plasmon Resonance Imaging (SPRI).

**Table 1:** Analytical data of the synthesized compounds

No.	Compound	Yield (%)	MW	[M + H]^+^	Retention time (min)
1	PhCH_2_SO_2_-d-Ser-Ala-Arg-NH-(CH_2_)_5_-NH_2_	47.3	570.7	571.8	13.36
2	2,4,6-TriiPrPhSO_2_-d-Ser-Ala-Arg-NH-(CH_2_)_5_-NH_2_	56.3	682.9	684.3	20.07
3	CH_3_PhSO_2_-d-Ser-Ala-Arg-NH-(CH_2_)_5_-NH_2_	54.6	494.6	495.3	13.99
4	PhCH_2_SO_2_-d-Ser-Gly-Arg-NH-(CH_2_)_5_-NH_2_	55.8	556.6	557.5	13.16
5	2,4,6-TriiPrPhSO_2_-d-Ser-Gly-Arg-NH-(CH_2_)_5_-NH_2_	49.7	668.8	669.6	21.23
6	CH_3_PhSO_2_-d-Ser-Gly-Arg-NH-(CH_2_)_5_-NH_2_	61.2	480.5	481.4	13.46
7	PhCH_2_SO_2_-d-Ser-NVa-Arg-NH-(CH_2_)_5_-NH_2_	56.7	598.7	599.9	14.08
8	2,4,6-TriiPrPhSO_2_-d-Ser-NVa-Arg-NH-(CH_2_)_5_-NH_2_	58.5	710.9	711.8	20.81
9	CH_3_PhSO_2_-d-Ser-NVa-Arg-NH-(CH_2_)_5_-NH_2_	59.7	522.6	523.5	14.94
10	PhCH_2_SO_2_-d-Ser-Gly-Arg-OH	53.5	472.5	473.5	13.59
11	2,4,6-TriiPrPhSO_2_-d-Ser-Gly-Arg-OH	56.7	584.7	585.8	21.24
12	CH_3_PhSO_2_-d-Ser-Gly-Arg-OH	55.7	396.4	397.5	22.70
13	PhCH_2_SO_2_-d-Ser-NVa-Arg-OH	54.6	514.6	515.7	15.33
14	2,4,6-TriiPrPhSO_2_-d-Ser-NVa-Arg-OH	63.1	626.8	627.6	22.87
15	CH_3_PhSO_2_-d-Ser-NVa-Arg-OH	52.1	438.5	439.4	16.17
16	H-d-Ser-NVa-Arg-NH-(CH_2_)_5_-NH_2_	62.4	444.5	445.9	8.57
17	H-d-Ser-Gly-Arg-NH-(CH_2_)_5_-NH_2_	63.2	402.5	403.9	8.74

## Experimental

### Reagents

Fmoc-Arg(Pbf)-OH (Fmoc = 9-fluorenylmethyloxycarbonyl, Pbf = pentamethyldihydrobenzofuran), chloranil, acetaldehyde, HOBt = 1-hydroxybenzotriazole, Fmoc-l-NVa-OH, Fmoc-l-Ala-OH, Fmoc-Gly-OH, TNBS = 2,4,6-trinitrobenzenesulfonic acid (1 % solution in DMF), methanesulfonyl chloride, α-toluenesulfonyl chloride and 2,4,6-triisopropylbenzenesulfonyl chloride were purchased from Fluka (Schnelldorf, Germany). Fmoc-d-Ser(*t*-Bu)-OH (*t*-Bu = *t*-butyl) and 2-chlorotrityl chloride resin were purchased from Merck (Novabiochem, Darmstadt, Germany). TFA = trifluoroacetic acid, DIPEA = diisopropylethylamine, DIC = diisopropylcarbodiimide, piperidine, TBTU = tetrafluoroborate salt of the *O*-(benzotriazol-1-yl)-*N*,*N*,*N*′,*N*′-tetramethyluronium tetrafluoroborate, NMP = 1-methyl-2-pyrrolidone and 1,5-diaminopentanetrityl resin were obtained from Iris Biotech GmbH (Marktrewitz, Germany). DCM = dichloromethane, DMF = dimethyl-formamide and methanol were the products of Chempur (Piekary Slaskie, Poland). DCM was used without further purification. DMF was distillated over ninhydrin and stored under molecular sieves 4A. HPLC solvent acetonitrile was purchased from Merck (Darmstadt, Germany). Urokinase, trypsin, kallikrein and Bzl-l-Arg-pNA^.^HCl (Bzl = benzyl) were purchased from Sigma (Schnelldorf, Germany). Plasmin, S-2444 (pyro-Glu-Gly-Arg-pNA^.^HCl), S-2238 (H-d-Phe-Pip-Arg-pNA), S-2251 (H-d-Val-Leu-Lys-pNA), S-2266 (H-d-Val-Leu-Arg-pNA^.^2HCl and S-2288 (H-d-Ile-Pro-Arg-pNA) were obtained from Chromogenix (Milano, Italy). Ac-Leu-Leu-Arg-H, cysteamine hydrochloride, *N*-(2-hydroxyethyl) piperazine-*N*′-(2-ethanesulfuric acid) (HEPES), *N*-ethyl-*N*′-(3-dimethylaminopropyl) carbodiimide (EDC) were purchased from Sigma (Steinheim, Germany). *N*-Hydroxysuccinimide (NHS) was obtained from Aldrich (Munich, Germany). Thrombin and phosphate buffered saline (PBS) were purchased from Lubelska Wytwórnia Szczepionek (Lublin, Poland). t-PA was obtained from Boehringer Ingelheim GmbH (Ingelheim, Germany). HBS-ES solution pH 7.4 (0.01 M HEPES, 0.15 M sodium chloride, 0.005 % Tween 20, 3 mM EDTA), acetic buffer pH 3.79–5.57, phosphate buffer pH 7.17–8.04, carbonate buffer pH 8.50–9.86 (Biomed, Lublin, Poland), photopolimer ELPEMER SD 2054, hydrophobic protective paint SD 2368 UV SG-DG (Peters, Kempen, Germany) were used as received. Aqueous solutions were prepared with MilliQ water (Simplicity^®^ MILLIPORE).

### Peptide Synthesis

The peptides shown in Table 1 were synthesized manually using the standard Fmoc-based strategy (Chan and White 2000). The Fmoc-Arg(Pbf)–OH was loaded on to the 2-chlorotrityl chloride resin with 2 M excess of DIPEA in DCM and in the case of 1,5-diaminopentane trityl resin using a molar ratio of amino acid/DIC/HOBt/resin 3:3:3:1 in DMF/NMP/DCM (1:1:1). Fmoc deprotection steps were carried out with 20 % (v/v) piperidine in DMF/NMP (1:1) for 15 min. The coupling reactions of Fmoc amino acids were performed in DMF/NMP/DCM (1:1:1) using a molar ratio of amino acid/DIC/HOBt/resin 3:3:3:1. In the case of the coupling of Fmoc-d-Ser(*t*-Bu)-OH molar ratio of amino acid/TBTU/HOBt/DIPEA/resin was 2:2:2:4:1. The sulfonyl chlorides were used as 5 M excess to resin and were dissolved in DCM with 10 M excess of DIPEA. The reactions were monitored with the Steward chloranil test (Vojkovski 1995) and with the TNBS test (Hancock and Battersby 1976). The cleavage from the resin was carried out with TFA/water (95/5). After 2.5 h stirring, the resin was filtered and washed with TFA. The combined filtrates were concentrated under reduced pressure. The crude peptide was precipitated and washed with cold diethyl ether, filtered, dissolved in water and lyophilized.

The Shimadzu LC-10A system (Shimadzu Europa GmbH, Duisburg, Germany) was used for analytical and semipreparatory HPLC (Phenomenex C18, Jupiter 90A, 4 micron, 250 × 10 mm; Phenomenex C18, Jupiter 300A, 5 micron, 250 × 4 mm; solvents: A, 0.1 % aqueous TFA; B, 0.1 % TFA in acetonitrile, gradient 0 % B to 100 % B in A in 30 min, flow rate 1 ml/min, monitored at 220 nm). The major peak fraction was pooled and lyophilized. The molecular weight determination was performed by mass spectrometry using a Bruker Daltonics Esquire 6000 (Bruker Daltonik GmbH, Leipzig, Germany) with electrospray ionization (ESI), (Table 1).

### Enzymatic Investigations

Determination of amidolytic activity was performed as previously described (Okada et al. 1988). The detailed description of the method is given below. The buffer and the enzyme solution included:

- tris buffer—0.6 ml (pH 8.8),

enzyme: urokinase (50 U/ml),

synthetic substrate: S-2444 (0.1 ml, 3 mM);

(b) tris buffer—0.5 ml (pH 8.4),

enzyme: thrombin (1 U/ml),

synthetic substrate: S-2238 (0.2 ml, 0.75 mM);

(c) tris buffer—0.5 ml (pH 7.4),

enzyme: plasmin (0.4 U/ml),

synthetic substrate: S-2251 (0.2 ml, 3 mM);

(d) borane buffer—0.5 ml (pH 7.5),

enzyme: trypsin (0.4 U/ml),

synthetic substrate: Bzl-l-Arg-pNA^.^HCl (0.2 ml, 8 mM);

(e) tris buffer—0.6 ml (pH 9.0),

enzyme: kallikrein (3 U/ml),

synthetic substrate: S-2266 (0.1 ml, 7.5 mM);

(f) tris buffer—0.6 ml (pH 8.4),

enzyme: t-PA (1.67 mg/ml),

synthetic substrate: S-2288 (0.1 ml, 10 mM).

Buffer and 0.1 ml of enzyme solution was added to 0.2 ml of examined compound dissolved in 0.15 M NaCl (**1**–**17**) (as control 0.15 M NaCl). The mixture was incubated for 3 min at 37 °C, then the synthetic substrate was added. After 20 min of incubation, the reaction was stopped by adding 0.1 ml of 50 % acetic acid, and the absorbance of the released *p*-nitroaniline was measured at 405 nm (Spekol 1300, AnalyticJena). Every value represents the average of the triplicate determination. IC_50_ value was considered as the concentration of the inhibitor, which decreased the absorbance at 405 nm by 50 %, compared with the absorbance measured under the same conditions without the inhibitor. K_i_ was calculated from IC_50_ based on Cheng–Prusoff equation (Cheng and Prusoff 1973). The results are given in Table 2.

**Table 2:** Inhibition of H-d-Ser-AA-Arg-OH on the amidolytic activity of enzymes

No.	K_i_ ± SD (μM)		
	Urokinase S-2444	Plasmin S-2251	Thrombin
1	n.i.	2.0 ± 0.16	n.i.
2	n.i.	90.9 ± 7.27	n.i.
3	n.i.	68.2 ± 5.45	n.i.
4	n.i.	58.2 ± 4.65	n.i.
5	44.6 ± 3.57	56.4 ± 4.51	n.i.
6	n.i.	46.4 ± 3.71	n.i.
7	n.i.	111.8 ± 8.95	n.i.
8	n.i.	52.7 ± 4.22	n.i.
9	n.i.	89.1 ± 7.13	n.i.
10	5.4 ± 0.43	102.7 ± 8.22	0.97 ± 0.08
11	15.8 ± 1.27	89.1 ± 7.13	n.i.
12	11.2 ± 0.89	10.0 ± 0.8	n.i.
13	n.i.	13.6 ± 1.09	0.82 ± 0.06
14	9.5 ± 0.76	120.0 ± 9.60	n.i.
15	n.i.	167.2 ± 13.38	n.i.
16	n.i.	137.3 ± 10.98	n.i.
17	n.i.	139.1 ± 11.13	n.i.
Ac-Leu-Leu-Arg-H (leupeptin)	IC_50_ = 42 μg/ml (Kawada and Umezawa 1995) n.i. (Saino et al. 1988)	IC_50_ = 5.8 μg/ml (Kawada and Umezawa 1995) IC_50_ = 10^−5^ M (Saino et al. 1988)	–
Ac-Leu-Leu-Arg-COCHO	–	4.9 μM (Lynas et al. 2001)	–

*n.i.* No cytotoxic effect was observed in maximum concentration (20 mM). The examined compound did not influence the enzymatic activity of trypsin, kallikrein and t-PA in maximum concentration (20 mM)

Our results were compared with the data obtained for Ac-Leu-Leu-Arg-H and Ac-Leu-Leu-Arg-COCHO (leupeptin—natural, microbial origin, inhibitor of proteinases and analog of leupeptin, respectively (Aoyagi et al. 1969)).

### Antitumor Activity

#### Tissue Culture

All studies were performed on MCF-7, MDA-MB-231 and DLD cells lines were purchased from American Type Culture Collection (Rockville, USA). The cells were maintained in DMEM supplemented with 5 % fetal bovine serum (FBS), 2 mmol/ml glutamine, 50 U/ml penicillin, 50 mg/ml streptomycin at 37 °C in a 5 % CO_2_ incubator.

#### Cytotoxicity Assay

The toxicity of the evaluated peptides was determined by the method of Plumb et al. (1989) in 10, 100, 250, 500 and 1,000 μM concentrations. MCF-7, MDA-MB-231 and DLD cells were maintained as described above. After 48 h of incubation of the cells with synthesized peptides, the medium was discarded and the cells were rinsed three times with phosphate buffered saline (PBS). The cells were then incubated for 4 h in 2 ml of PBS with 50 ml of MTT (5 mg/ml). After removal of the medium, the cells were lysed in 200 ml of DMSO with 20 ml of Sorensen’s buffer (0.1 M glycine with 0.1 M NaCl, pH 10.5). The absorbance was measured at 570 nm. The cytotoxic activity of synthesized peptides was calculated as percentage of nonviable cells and the IC_50_ value was estimated from logarithm curves as shown in Table 3.

**Table 3:** The nonviability of MDA cells treated for 24 h with different concentrations of the synthesised peptides

Compounds	IC_50_ (μM) (% of control ± 2)	
	MDA	DLD
1	480	460
2	480	390
3	570	390
4	580	320
5	510	150
6	450	260
7	680	340
8	660	330
9	620	50
10	640	5
11	570	110
12	620	230
13	650	180
14	590	220
15	650	270
16	490	260
17	510	250
Leupeptin	580	250

### Hemolytic Activity

Pig’s fresh red blood cells (p-RBC) were washed three times with PBS (35 M phosphate buffer/0.15 mM NaCl, pH 7.4) and were centrifugated at 1,000*g* for 10 min to remove plasma and the buffy coat. The various concentrations of peptides (100, 250, 500 and 1,000 μg/ml) were incubated with the erythrocyte suspension for 1 h at 37 °C (the final erythrocyte concentration was 5 % v/v). After the centrifugation (1,000*g* for 10 min), 100 μl of the supernatant was transferred into sterilized 96-well plates, where hemoglobin release was monitored with the use of the Infinite M200 plate reader (TECAN, Salzburg, Austria) by measuring the absorbance at 414 nm. Zero hemolysis (blank), hemolysis with Ac-Leu-Leu-Arg-H as reference compound for synthesised peptides and 100 % hemolysis which consisted of p-RBC suspended in PBS and 0.1 % Triton-X-100 were determined respectively. The percentage of hemolysis was calculated with the following formula: hemolysis (%) = (Abs_414 nm_ in the peptide solution in PBS/Abs_414nm_ in 0.1 % Triton-X-100 in PBS) × 100.

### SPRI Investigation

#### Chip Preparation

Gold chips were manufactured as described in previous papers (Gorodkiewicz 2009; Gorodkiewicz and Regulska 2010; Gorodkiewicz et al. 2011). The gold surface of the chip was covered with photopolymer and hydrophobic paint. 9 × 12 free gold surfaces were obtained. Using this chip, nine different solutions can be simultaneously measured without mixing the tested solutions. Twelve single SPRI measurements can be performed from one solution.

#### Inhibitor Immobilization

Chips were rinsed with ethanol and water and dried under a stream of nitrogen. They were then immersed in 20 mM cysteamine ethanolic solutions for at least 2 h. The chips were then rinsed with ethanol and water and dried under a stream of nitrogen.

**Fig. 1:**
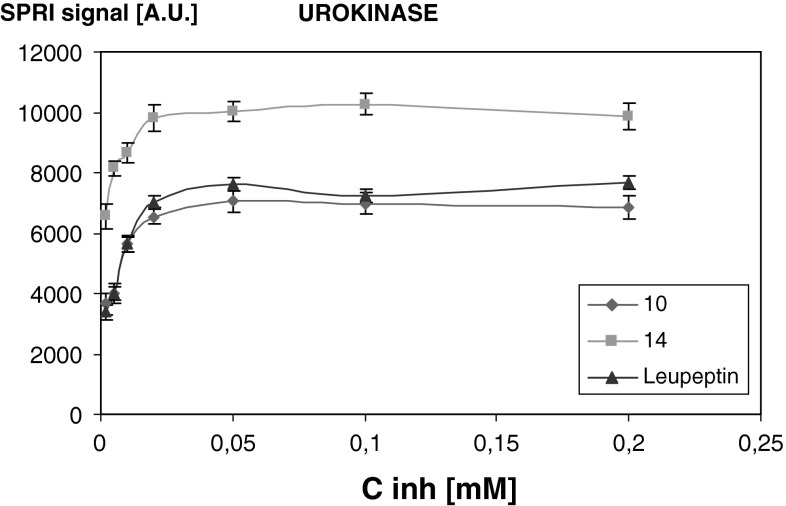
Dependence of SPRI signal (a.u.) of urokinase-inhibitor complex on inhibitor concentration. Urokinase concentration 1 ng/ml

**Fig. 2:**
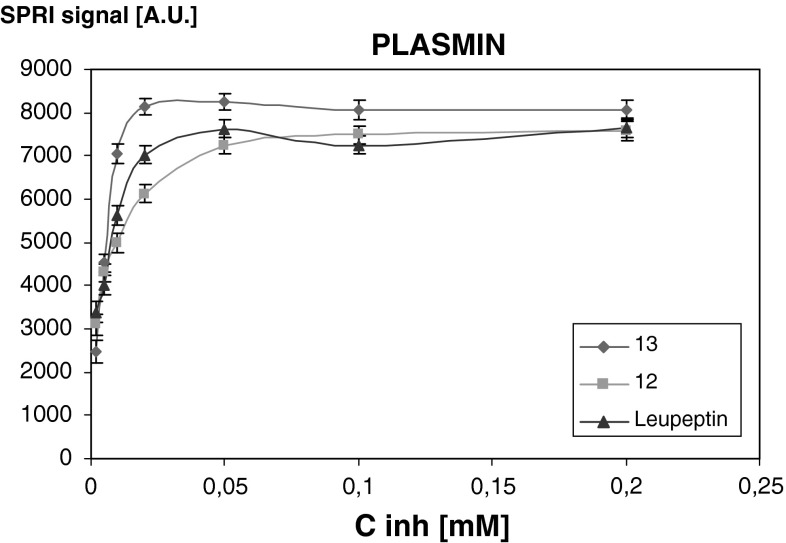
Dependence of SPRI signal (a.u.) of plasmin-inhibitor complex on inhibitor concentration. Plasmin concentration 1 ng/ml

Among the synthesised compounds, four were selected for the examination of the interaction between the enzyme and the inhibitor. They were acids of peptides 10, 12, 13 and 14. Acids can be bound to a gold surface via cysteamine. A series of concentrations 0.002, 0.005, 0.01, 0.02, 0.05, 0.1 and 0.2 mM was used for each peptide. The concentration of the enzymes was constant 1 ng/ml. Inhibitor solution, activated with NHS (50 mM) and EDC (200 mM) was placed on the cysteamine-modified surface, and incubated at 37 °C for 1 h (Gorodkiewicz 2009; Gorodkiewicz and Regulska 2010; Gorodkiewicz et al. 2011). The chip was then treated with the urokinase or plasmin solution for 10 min, rinsed with HBS–ES buffer and dried. Finally, the SPRI measurement was performed. Each solution was put on two measuring fields (each consisting of 12 measuring points); thus 24 replicates were done in each case. The results are given in Figs. 1 and 2.

#### SPRI Measurements

SPRI measurements with an application of an enzyme array were performed as described in previous papers (Gorodkiewicz 2009; Gorodkiewicz and Regulska 2010; Gorodkiewicz et al. 2011). Briefly, the measurements were performed at a fixed angle of incident light and the reflectivity was simultaneously measured across an entire chip surface. The contrast values obtained for all pixels across a particular sample single spot were integrated. Thus the SPRI signal was integrated over the single spot area. Background correction was applied. NIH ImageJ version 1.42 software was used to evaluate the SPRI images in 2D form. The signal was measured twice on the basis of registered images, after immobilisation of enzymes and then after interaction inhibitors. The SPRI signal, which is proportional to coupled enzymes, was obtained from subtraction between the signal before and after interaction with enzymes for each spot separately.

## Results and Discussion

### Inhibition of the Serine Proteases

The examined compound did not influence the enzymatic activity of trypsin, kallikrein and t-PA.

We expected that the use of the specific tripeptide sequence towards urokinase (D-Ser-AA-Arg) would cause some selectivity (Ke et al. 1997). According to the obtained results, only five compounds inhibited urokinase activity but none of them were selective. The most active inhibitor of u-PA was **10** PhCH_2_-SO_2_-d-Ser-Gly-Arg-OH with K_i_ value 5.4 μM. H-d-Ser-Gly-Arg-OH and H-d-Ser-Ala-Arg-OH were previously examined (Markowska et al. 2008) and they were the most active inhibitors of urokinase with IC_50_ value of 1 mM and 2 mM respectively (K_i_ = 90 μM and 180 μM, author’s unpublished data). In 2010 we synthesised *N*-sulfonylamide peptides with alanine as P2 (Markowska et al. 2010). *α*-Tolyl- and *p*-tolylsulfonylamides of the examined tripeptides were active towards urokinase. The compound 2,4,6-triisopropylphenylsulfonyl-d-Ser-Ala-Arg-OH was the most selective and we chose 2,4,6-triisopropylphenylsulfonyl fragment to the present research. In current work only compound **14** 2,4,6-triiPrPhSO_2_-d-Ser-NVa-Arg-OH had some activity towards urokinase, but in this case it was not selective. Only amide of arginine **5** 2,4,6-triiPrPhSO_2_-d-Ser-NVa-Arg-NH-(CH_2_)_5_-NH_2_ were active urokinase inhibitors with K_i_ value 44.6 μM.

All of the synthesised peptides were inhibitors of plasmin. The compound **1** PhCH_2_SO_2_-d-Ser-Ala-Arg-NH-(CH_2_)_5_-NH_2_ was the most active plasmin inhibitor with K_i_ value of 2 μM in this series of peptides. Compound **1** contains a fragment of pentamethylenediamine (cadaverine) as amide arginine residue in its structure which seems to have significant influence on the activity and selectivity. The moiety of cadaverine is the form of decarboxylated derivative of lysine. In literature, there are peptides with C-terminus lysine amide and also amide residue of lysine substituted with cadaverine, as inhibitors of plasmin (Markowska et al. 2007; Midura-Nowaczek et al. 2006). This kind of compounds was designed to be similar to natural substrate sequence hydrolysed by plasmin.

The most active inhibitors of thrombin in whole series of compounds were **10** PhCH_2_SO_2_-d-Ser-Gly-Arg-OH and **13** PhCH_2_SO_2_-d-Ser-NVa-Arg-OH with K_i_ value 0.97 and 0.82 μM.

### Antitumor Activity of Synthesised Peptides

The examined compounds did not influence MCF-7 cancer cells. It was found that the proteolytic activity of uPA is closely connected with cell-surface events at the breast cancer cell. MCF-7 has low uPAR/uPA-expressing and low plasminogen-binding, whereas MDA-MB-231 has high uPAR/uPA-expressing and high plasminogen-binding (Stillfried et al. 2007). Thus, the influence of the synthesized compounds on the cytotoxic effect of MCF-7 cells could be insignificant.

The peptides **1–17** and the examined natural inhibitor of proteinases leupeptin were comparably cytotoxic to MDA-MB-231. The most interesting peptide was compound **10** with an IC_50_ values of 5 μM for DLD cells, which was the most active inhibitor of urokinase in the amidolytic test.

### Hemolytic Activity

The results indicated that the concentration up to 1,000 μg/ml of the synthesized peptides did not lyse porcine erythrocytes.

### The Effect of Newly Synthesised Inhibitor Concentration on the SPRI Signals of Plasmin and Urokinase

The first aim of this investigation was the qualitative studies of the interaction between the potential inhibitor and the enzyme. The second aim was to find an optimal inhibitor concentration for the interaction with the enzymes.

The positive SPRI signal was obtained for urokinase after the interaction with inhibitors **10** and **14**, as well as for plasmin with inhibitors **12** and **13**. The obtained curves for compounds **10**, **12**, **13** and **14** were a Langmuir isotherm type, with the plateau of the signal above 0.02 mM. Leupeptin was used as a reference inhibitor for plasmin and urokinase. The newly synthesised inhibitors act in a similar way as leupeptin.

## Conclusion

The most active compound towards urokinase was PhCH_2_SO_2_-d-Ser-Gly-Arg-OH with K_i_ value 5.4 μM and the most active compound toward thrombin was PhCH_2_SO_2_-d-Ser-NVa-Arg-OH with K_i_ value 0.82 μM. The peptides were nontoxic against porcine erythrocytes in vitro. PhCH_2_SO_2_-d-Ser-Gly-Arg-OH compound showed cytotoxic effect against DLD cell lines with IC_50_ values of 5 μM. Some of the synthesised compounds bind to urokinase and plasmin in 0.05 mM concentration evaluated by the Surface Plasmon Resonance Imaging (SPRI) sensor.
